# Large Unilateral Pleural Effusion as an Initial Manifestation of Systemic Lupus Erythematosus

**DOI:** 10.1002/ccr3.72704

**Published:** 2026-05-13

**Authors:** Satya Rijal, Aishwarya Holi, Prakash Khanal

**Affiliations:** ^1^ Department of Internal Medicine Michigan State University College of Human Medicine East Lansing Michigan USA; ^2^ Internal Medicine Residency Program University of Michigan Health–Sparrow Lansing Michigan USA; ^3^ Michigan State University College of Human Medicine East Lansing Michigan USA

**Keywords:** autoimmune disease, heart failure mimic, serositis, systemic lupus erythematosus, unilateral pleural effusion

## Abstract

Heart failure and unilateral pleural effusion may rarely represent initial manifestations of SLE in adults. Diagnostic evaluation is often challenging due to overlap with common conditions such as congestive heart failure. Early recognition and timely management are crucial for preventing disease progression and achieving optimal clinical outcomes.

## 1 Introduction

Systemic lupus erythematosus (SLE) is a multisystem autoimmune disorder that most commonly affects young women [1]. While pleuritis is the most common thoracic manifestation, occurring in 17%–60% of patients, pleural effusions are reported in up to 50% of SLE patients and are usually bilateral [2, 3]. Large unilateral pleural effusion as an initial sign are rare and may be misdiagnosed as heart failure, infection, or cancer, especially in older or male patients [2, 4, 5]. Unilateral effusions are typically exudative, may be associated with elevated C‐reactive protein, and often indicate active or severe disease, sometimes with cardiac involvement [4, 5]. Pleuropulmonary symptoms—including lupus pleuritis, acute pneumonitis, shrinking lung syndrome, interstitial lung disease, and diffuse alveolar hemorrhage—are recognized diagnostic features of SLE [4, 5, 6, 7]. Although lupus pleuritis accounts for about half of SLE‐related effusions, large unilateral effusions are rare and can be challenging to diagnose. Early recognition is crucial for preventing delays in diagnosis and initiating prompt immunosuppressive treatment [4, 5, 6, 7].

We report a rare case of a previously healthy 60‐year‐old male who presented with worsening shortness of breath, fatigue, and generalized swelling, initially misdiagnosed and treated as decompensated heart failure due to a large unilateral pleural effusion. Despite diuretic therapy, symptoms persisted. Laboratory evaluation revealed normocytic anemia, elevated erythrocyte sedimentation rate (ESR) and C‐reactive protein (CRP), mild renal dysfunction, and mildly elevated brain natriuretic peptide (BNP). Pleural fluid analysis confirmed an exudative effusion with the presence of lupus erythematosus (LE) cells. Autoimmune serology demonstrated positive antinuclear antibody (ANA) (speckled pattern), anti‐double‐stranded DNA (anti‐dsDNA) antibodies, anti‐Smith (anti‐Sm) antibodies, and low complement levels. Initiation of corticosteroids and immunosuppressive therapy (hydroxychloroquine and mycophenolate mofetil) led to rapid clinical improvement, with complete resolution of pleural effusion at 3 months. This case highlights the importance of considering SLE in unexplained unilateral pleural effusions to avoid misdiagnosis and initiate timely immunosuppressive therapy.

## 2 Case History

A 60‐year‐old Caucasian Male with a history of hypothyroidism presented to the emergency department with a 2‐week history of progressive shortness of breath, orthopnea, nocturnal dry cough, and bilateral lower extremity edema, and 3 days of progressive shortness of breath at rest. Three days before admission, dyspnea occurred at rest. He described his chest pain as typically sharp and stabbing, starting 2–3 days ago, which was often exacerbated by deep breaths, coughing, sneezing, or laughing. He denied fevers, rash, arthralgias, oral ulcers, or neurologic symptoms. Past medical history included hypothyroidism. Family history was negative for autoimmune diseases.

## 3 Physical Examination

Upon admission, his vital signs included a blood pressure of 140/90 mmHg, a heart rate of 110 beats per minute, a respiratory rate of 28 breaths/min, and an oxygen saturation of 88% on room air. The patient exhibited significant respiratory compromise, requiring oxygen supplementation via nasal cannula. Physical examination revealed substantial respiratory distress requiring 4 L of oxygen, bilateral pitting edema extending to the thighs, and diminished breath sounds at the left lung base.

There was no evidence of cardiac murmurs, jugular venous distension, or hepatomegaly. Skin examination did not reveal any rashes, photosensitivity, or oral ulcers. Musculoskeletal examination was initially unremarkable for synovitis or joint deformities.

During the third day of hospitalization, the clinical symptoms of generalized joint pains and myalgias without overt arthritis became apparent.

## 4 Differential Diagnosis

The differential diagnosis of unilateral pleural effusion is broad, including heart failure, infections, malignancy, benign asbestos pleural effusion, and systemic lupus erythematosus. Heart failure can cause pleural effusions, often bilateral, but it can also present atypically as a large unilateral effusion without other typical signs of heart failure. Empyema or parapneumonic effusion (bacterial, usually associated with pneumonia), tuberculosis (consider with elevated pleural fluid ADA), and less commonly, Salmonella are also causes. In older patients, a significant concern is that unilateral pleural effusion often appears as an exudate. Benign asbestos pleural effusion is possible in patients with a history of asbestos exposure, although it may mimic other conditions before a correct diagnosis is made. Systemic lupus erythematosus (SLE) is an autoimmune disease where pleural effusion, often unilateral and exudative, can be an initial or dominant manifestation. Due to its atypical presentations, SLE can be difficult to diagnose in older individuals.

## 5 Investigations

Initial laboratory investigations showed normocytic normochromic anemia, a high erythrocyte sedimentation rate (ESR) of 80 mm/h, elevated C‐reactive protein (CRP) at 17.38, Hemoglobin 9.5 g/dL (normal: 13–17 g/dL), Mean Corpuscular Volume (MCV) was 90 fL (normal: 80–100 fL), Mean Corpuscular Hemoglobin Concentration (MCHC) was 33% (normal: 32%–36%), slightly raised creatinine of 1.9 with a urine albumin‐to‐creatinine ratio (ACR) of 486.71 mg/g, and routine liver function tests. Brain Natriuretic Peptide (BNP) levels were mildly elevated at 150 pg/mL (normal < 100 pg/mL) (Table 1).

**TABLE 1:** Patient's laboratory values at presentation.

Test	Patient result	Reference range	Unit	Notes
Hemoglobin (Hb)	9.5	13.0–17.0	g/dL	↓ Low
Mean corpuscular volume (MCV)	90	80–100	fL	Normal
Mean corpuscular hemoglobin concentration (MCHC)	33	32–36	g/dL	Normal
Erythrocyte sedimentation rate (ESR)	80	< 50	mm/h	↑ High
C‐reactive protein (CRP)	17.38	< 0.9	mg/dL	↑ High
Creatinine (serum)	1.9	0.7–1.3	mg/dL	↑ High
Urine albumin‐to‐creatinine ratio (ACR)	486.71	< 30	mg/g	↑ High
Alanine aminotransferase (ALT)	25	0–45	IU/L	Normal
Aspartate aminotransferase (AST)	28	0–35	IU/L	Normal
Brain natriuretic peptide (BNP)	150	< 100	pg/mL	↑ High

**FIGURE 1:**
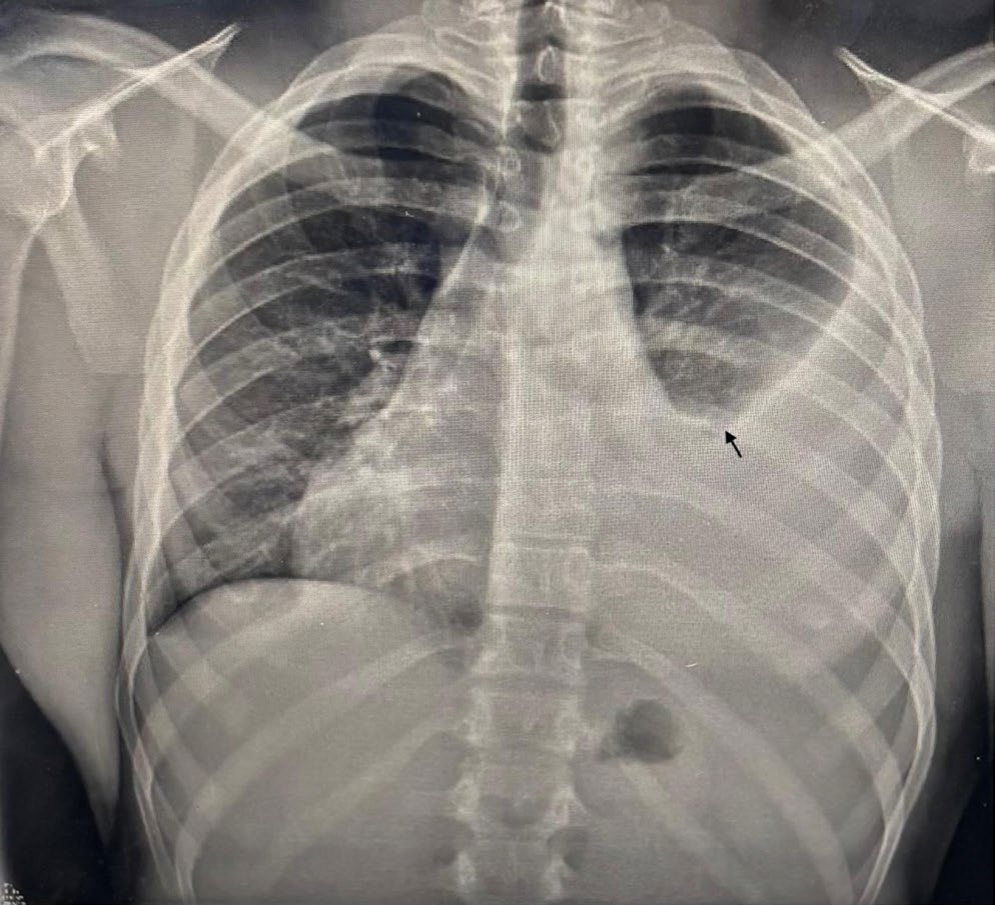
Chest X‐Ray (anterior and posterior view) showing rightward displacement of the cardiac silhouette and a black arrow showing a large left unilateral pleural effusion.

**FIGURE 2:**
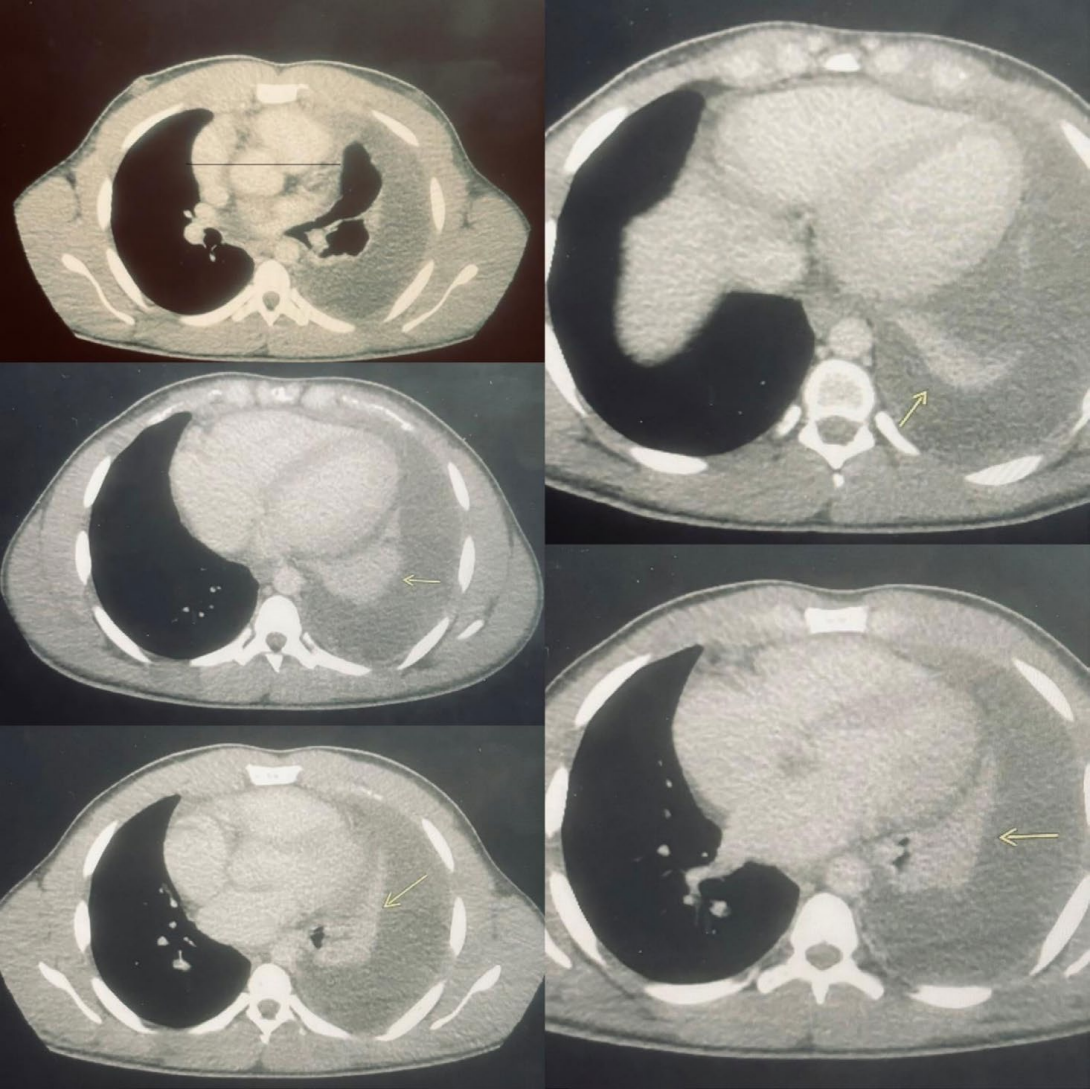
Computed tomography (CT) scan of the chest with contrast, with a transverse line showing a borderline enlarged heart, and additional images, including views of the lung bases with yellow arrows showing a large left pleural effusion, loss of upper left lung volume indicating collapsed portions of the left upper lobe, and a compression atelectasis involving much of the left lower lobe.

An electrocardiogram (ECG) revealed sinus tachycardia. A chest X‐ray showed massive unilateral pleural effusion with blunting of the left costophrenic angle and obscured cardiac border on the left side (Figure 1). A CT scan confirmed a left‐sided large effusion, which further supported the initial diagnosis of heart failure (Figure 2).

He was admitted to an inpatient service and started on intravenous diuretics and oxygen therapy. Given the lack of significant response to standard heart failure treatment, including aggressive diuresis for 48 h, and the disproportionate size of the effusions relative to the mild BNP elevation, further investigations were pursued. Echocardiography revealed a normal left ventricular ejection fraction (LVEF 60%) without regional wall motion abnormalities, significant valvular abnormalities, and no pericardial effusion, effectively ruling out primary cardiac failure. Thoracentesis was performed on the left side, yielding 1.5 L from the left pleural space. Pleural fluid analysis showed an exudative effusion (protein fluid/serum ratio of 0.6 [> 0.5], LDH fluid/serum ratio of 0.8 [> 0.6]) with a high lymphocyte count meeting Light's criterion for exudate. Cytology revealed the presence of Lupus Erythematosus (LE) cells, which were negative for malignancy. Tests for *Mycobacterium tuberculosis* were negative. Subsequent autoimmune workup showed a positive Antinuclear Antibody (ANA) with a speckled pattern (titer 1:1280), positive anti‐double‐stranded DNA (anti‐dsDNA) antibodies (250 IU/mL; normal < 25 IU/mL), and significantly decreased complement levels (C3 45 mg/dL, normal 90–180 mg/dL; C4 8 mg/dL, normal 10–40 mg/dL). Anti‐Sm antibodies were positive, but anti‐RNP antibodies were negative (as mentioned in Table 2). Based on these findings, the patient met the revised American College of Rheumatology (ACR) classification criteria for Systemic Lupus Erythematosus [5].

**TABLE 2:** Summary of thoracentesis and autoimmune workup results.

Test	Normal	Patient result	Interpretation
ANA	< 1:80	1:1280 speckled	Strongly positive
Anti‐dsDNA	< 25 IU/mL	250 IU/mL	Highly elevated
Anti‐Sm	Negative	Positive	SLE‐specific
Anti‐RNP	Negative	Negative	Not detected
Complement C3	90–180 mg/dL	45 mg/dL	Low
Complement C4	10–40 mg/dL	8 mg/dL	Low
Pleural fluid LE Cells	Negative	Positive	Supports SLE
Pleural fluid exudate	N/A	Exudative	Meets Light's criteria

Abbreviations: ANA, anti‐nuclear antibody; Anti‐dsDNA, anti‐double‐stranded antibody; Anti‐RNP, anti‐ribonuclear protein antibody; Ant‐Sm, anti‐Smith antibody.

## 6 Treatment

The initial misdiagnosis of heart failure delayed appropriate treatment. Once the diagnosis of SLE was established, the patient was promptly initiated on immunosuppressive therapy. He received intravenous methylprednisolone 1 mg/kg/day for 3 days, followed by oral prednisone 60 mg daily. Hydroxychloroquine 200 mg twice daily was also started. Given the severity of his presentation and the risk of other organ involvement, mycophenolate mofetil (MMF) 1 g twice daily was added for long‐term disease control. The diuretics were gradually tapered as the effusions began to resolve. The patient received supportive care, including oxygen therapy, pain management for pleuritic pain, and nutritional support.

## 7 Results (Outcomes and Follow‐Up)

The patient's dyspnea significantly improved within 72 h of initiating corticosteroid therapy. Follow‐up chest X‐rays showed remarkable resolution of the left pleural effusion over the next week. His lower extremity edema also subsided. He was discharged from the hospital after 10 days, with a plan for outpatient follow‐up with a rheumatologist. At his 2‐month follow‐up, he reported complete resolution of dyspnea and fatigue. The physical examination was unremarkable, and repeated laboratory tests showed normalization of complement levels and a decrease in anti‐dsDNA antibody titers (as mentioned in Table 3). After 2 months, he showed significant clinical improvement in pleural effusion.

**TABLE 3:** Follow‐up laboratory findings at 2 months.

Test	Patient initial	Follow‐up	Interpretation
Anti‐dsDNA	250 IU/mL	45 IU/mL	Decreased
C3	45 mg/dL	100 mg/dL	Normalized
C4	8 mg/dL	25 mg/dL	Normalized
ESR	80 mm/h	15 mm/h	Normalized
CRP	17.38 mg/dL	0.5 mg/dL	Normalized

At his 6‐month follow‐up, he continues a tapering dose of prednisone, hydroxychloroquine, and mycophenolate mofetil, with no recurrence of pleural effusions or other lupus flares. His quality of life has significantly improved, and he has resumed daily activities.

## 8 Discussion

Pleural involvement is the most common thoracic manifestation of systemic lupus erythematosus (SLE), with lupus pleuritis being the leading cause of pulmonary symptoms in affected patients [1]. Although pleural effusions often accompany other systemic signs of active disease, recent reports show that older adults can present with large pleural effusions early in the disease, even without typical skin or kidney findings [3, 4]. Our patient's presentation aligns with these observations and emphasizes the importance of considering autoimmune disease when evaluating new‐onset pleural effusion in the elderly.

The initial diagnostic challenge in this case stemmed from overlap with heart failure, a much more common cause of pleural effusion in older populations. Mild BNP elevation and dyspnea made decompensated heart failure a plausible early diagnosis. However, several inconsistencies—including unilateral effusion distribution, mild cardiomegaly, a normal echocardiogram, and failure to improve with diuretics—prompted a reassessment of the working diagnosis. While heart failure can sometimes cause unilateral effusions, they are usually bilateral and transudative. Furthermore, the misclassification of unilateral effusions is well documented, highlighting the need to re‐evaluate when clinical features do not match the expected pattern [3, 4].

Pleural fluid analysis was key to clarifying the diagnosis. The fluid was exudative and lymphocyte‐predominant, with high protein, normal glucose, and pH > 7.30—findings more consistent with lupus pleuritis than with heart failure, infection, or malignancy [4]. Tuberculosis was ruled out by low Adenosine Deaminase (ADA) levels and a negative interferon‐gamma release assay, and imaging did not suggest malignancy. The presence of LE cells, along with positive ANA, elevated anti‐dsDNA and anti‐Sm antibodies, and significant hypocomplementemia strongly supported an SLE‐related effusion. The pleural fluid–to–serum ANA ratio indicated local antibody production, while the absence of rheumatoid factor and anti‐CCP helped differentiate lupus pleuritis from rheumatoid pleuritis, which typically shows very low glucose and high LDH levels [4].

Pleural involvement often indicates increased SLE disease activity and is linked to broader systemic inflammation. Studies reveal that patients with lupus pleuritis often have higher SLEDAI‐2K scores, hematologic issues like anemia and thrombocytopenia, and higher rates of anti‐RNP, anti‐Sm, anti‐La/SSB, chromatin, and antiphospholipid antibodies [5, 6, 7]. Symptoms may include fever, weight loss, lymphadenopathy, nephritis, or pericarditis, although skin findings might be less noticeable in late‐onset disease. These points emphasize that pleural effusions can signal wider systemic involvement [7].

Cardiac assessment remains crucial, as lupus myocarditis can present with new heart failure and may mimic or accompany lung and pleural issues. Although rare, it is often underdiagnosed; autopsy studies report myocarditis in up to 57% of SLE patients without prior suspicion [8, 9]. In our patient, the lack of imaging evidence of myocarditis was reassuring, but ongoing dyspnea and elevated BNP highlighted the need for a detailed cardiac evaluation.

Managing lupus pleuritis is usually very effective, with most patients responding quickly to systemic corticosteroids. Additional immunosuppressive treatments—such as hydroxychloroquine, mycophenolate mofetil, azathioprine, or cyclophosphamide—are used in more active or multisystem cases [9]. Refractory cases have been successfully treated with rituximab. In the Phase II trial, newer biologics, such as telitacicept, showed particular benefit in patients with moderate‐to‐severe, serologically active SLE—especially those with high anti‐dsDNA titers, low complement levels, and an inadequate response to standard immunosuppressive therapy [10]. Invasive procedures, including pleurodesis or decortication, are rare and reserved for recurrent or fibrothorax‐associated effusions [10].

## 9 Conclusion

Large unilateral pleural effusion is an uncommon presentation of systemic lupus erythematosus and may resemble more common causes such as heart failure. When pleural fluid is exudative and cardiac or infectious etiologies are excluded, clinicians should consider lupus pleuritis—even without classic SLE features. A systematic approach that includes pleural fluid analysis, autoimmune serology, and careful exclusion of other causes is essential for accurate diagnosis. Early recognition is crucial because lupus pleuritis typically responds well to corticosteroids and immunosuppressive therapy, helping prevent further thoracic or systemic complications. This case underscores the importance of including SLE in the differential diagnosis of isolated pleural effusion and performing a comprehensive evaluation to guide effective management.

## Author Contributions

**Satya Rijal:** conceptualization, data curation, formal analysis, funding acquisition, investigation, methodology, project administration, resources, software, validation, visualization, writing – original draft, writing – review and editing. **Aishwarya Holi:** conceptualization, data curation, formal analysis, funding acquisition, investigation, methodology, project administration, resources, software, validation, visualization, writing – original draft, writing – review and editing. **Prakash Khanal:** conceptualization, data curation, formal analysis, funding acquisition, investigation, methodology, project administration, resources, software, supervision, validation, visualization, writing – original draft, writing – review and editing.

## Funding

This work was supported by the Michigan State University College of Medicine, Michigan State University.

## Consent

Written informed consent was obtained from the patient to publish this report. This case report has been completely anonymized.

## Conflicts of Interest

The authors declare no conflicts of interest.

## Data Availability

The authors have nothing to report.
